# Terahertz Absorber with Graphene Enhanced Polymer Hemispheres Array

**DOI:** 10.3390/nano11102494

**Published:** 2021-09-24

**Authors:** Alesia Paddubskaya, Nadzeya Valynets, Sergey Maksimenko, Mukesh Kumar, Marian Baah, Markku Pekkarinen, Yuri Svirko, Gintaras Valušis, Polina Kuzhir

**Affiliations:** 1 Laboratory of Nanoelectromagnetics, Institute for Nuclear Problems of Belarusian State University, Bobruiskaya Str. 11, 220006 Minsk, Belarus; paddubskaya@gmail.com (A.P.); Nadezhda.Volynets@gmail.com (N.V.); sergey.maksimenko@gmail.com (S.M.); 2 Institute of Photonics, Department of Physics and Mathematics, University of Eastern Finland, Yliopistokatu 7, 80101 Joensuu, Finland; mkkhatri32@gmail.com (M.K.); marian.baah@uef.fi (M.B.); markku.pekkarinen@uef.fi (M.P.); yuri.svirko@uef.fi (Y.S.); 3 Center for Physical Sciences and Technology, Saulėtekio Av. 3, 10257 Vilnius, Lithuania; gintaras.valusis@ftmc.lt

**Keywords:** graphene, metasurface, terahertz, absorption, electromagnetic wave

## Abstract

We propose an original technique for the fabrication of terahertz (THz) metasurfaces comprising a 3D printed regular array of polymer hemispheres covered with a thin conductive layer. We demonstrate that the deposition of a thin metal layer onto polymer hemispheres suppresses the THz reflectivity to almost zero, while the frequency range of such a suppression can be considerably broadened by enhancing the structure with graphene. Scaling up of the proposed technique makes it possible to tailor the electromagnetic responses of metasurfaces and allows for the fabrication of various components of THz photonics.

## 1. Introduction

Broadband and frequency-tunable electromagnetic (EM) absorbers are widely used for the fabrication of a variety of EM components including polarizers, filters, attenuators and other devices of the microwave and THz photonics [1,2,3,4]. Sculpturing of conductive surfaces is one of the most developed techniques to enhance absorption at the microwave and THz frequencies [5]. Conventional EM absorbers relies on metal surface covered with subwavelength pyramid-like structures organized in a square or hexagonal lattice [6]. In such a structure, enhancement of the microwave absorption is achieved via “smoothing” the metal-vacuum interface when the incident EM wave “feels” the gradual change of the impedance from vacuum to the material one. However, efficient absorption requires pyramids taller than the quarter of the wavelength [7]. That is, the height of the pyramidal structure on the surface should exceed 2 m to efficiently absorb radiation at the frequency of 30 MHz or should have the height of 10 cm to absorb EM waves in the frequency range of 8–40 GHz. The fabrication of such structures for the mega- and gigahertz range is well documented and their cost rapidly decreases due to development of the 3D printing technology [8]. However, in the THz range, the height of the pyramidal structure should be in the sub-millimeter range, i.e., a costly and time-consuming lithography techniques are needed to control the shape of the surface corrugations [9].

The above problems can be partially solved by replacing sculptured metal structure with graphene or other ultrathin carbon films possessing high absorption ability [10] in the THz range. High conductivity [11], record carrier mobility of graphene [12] combined with optical transparency at room temperature [13] and the pronounced sensitivity of its electronic properties to environment and external forces [14] allow one to use graphene-based absorbers for detecting tiny variations of the EM field, e.g., for using them in bolometers and THz sensors.

Placing not-structured graphene sheet onto flat dielectric substrate supported by metallic back reflectors [15,16] is a well-established approach to achieve perfect absorption of EM radiation at particular frequency. There have also been several techniques developed for incorporating graphene into semiconductor and metal 3D patterns [17,18,19,20] and for using multilayer graphene islands or nanoribbons [21,22], which can be synthesized on the dielectric surfaces via a sacrificial metal layer.

However, the intrinsic planar structure of graphene imposes severe limitations on the using it for sensing [23,24]. This is due to the fact that the EM response of graphene-based sensing devices shows pronounced frequency dependence, which is governed solely by the graphene insulating support. Broadband EM absorbers require either patterning of graphene, i.e., making graphene-based metamaterial or using a multilayers approach. The fabrication of graphene-based metamaterial [25] involves time-consuming electron beam lithography and etching that make scaling up this approach a rather difficult task. Moreover, the EM response of such a metamaterial will be dependent not only on the graphene quality and doping level, but also on the quality of the graphene patterning process. The multilayer approach allows decreasing the thickness of the absorber by using a quarter wavelength thick Salisbury screen [26], which, however, suppresses the level of absorption substantially.

In this paper, we show that these difficulties can be overcome by enhancing the 3D metasurface with the flat graphene sheet. We propose a simple and scalable technique for fabrication of such graphene enhanced metasurfaces combining 3D printing, metal deposition through thermal evaporation, or magnetron sputtering and conventional graphene transfer processes.

We choose the periodic array of polymer hemispheres as a metasurface scaffold since it provides facile matching of the medium/air impedances to approach suppressing the reflection of EM radiation. The multiple reflections in between individual hemispheres couple the incident wave to the surface ones making the metasurface highly absorptive at certain frequencies, which depend on the diameter of hemisphere and periodicity of the array. Covering of the polymer corrugated surface with thin layer of metal (imaginary part of permittivity Im ε ≫ 1), which thickness *l* is much smaller that the skin depth and the wavelength *l* √ε ≪ λ, should lead to substructional absorption of THz radiation [27].

We demonstrate that graphene enhanced 3D printed metasurfaces are capable to achieve almost zero reflectance and >70% absorptance at the spectral range spanning from 200 GHz to 1 THz and can exhibit nearly perfect absorption at the frequencies of 0.8–1 THz.

## 2. Electromagnetic Modelling

In the course of the optimizing geometry of the structure in terms of the hemisphere diameter and the array periodicity, we performed numerical simulation of the EM response of the silica/polymer hemispheres/Ni and silica/polymer hemispheres/Ni/graphene metasurfaces in the CST Studio environment.

**Figure 1:**
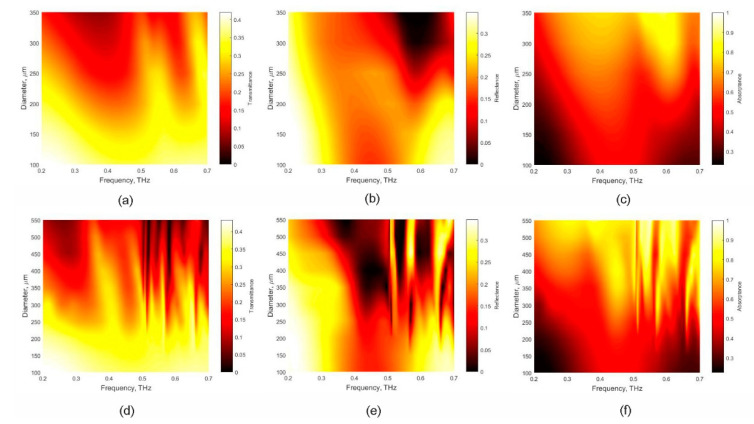
Transmittance (*T*), reflectance (*R*), and absorptance (*A* = 1 − *T* − *R*) of multilayer system contained the array of polymer hemispheres covered with Ni layer (30 nm thick) and deposed on SiO_2_ substrate versus hemispheres diameter and frequency. The calculations were performed for the structure organized in the square lattice with 400 μm (**a**–**c**) and 600 μm (**d**–**f**) period, respectively. The following parameters were used in the simulation: the polymer dielectric permittivity was 2.3; the dielectric permittivity of SiO_2_ substrate was set 2.7.

Figure 1 shows the transmittance (*T*), reflectance (*R*), and absorptance (*A*) calculated for the square lattice array of polymer hemispheres covered with thin Ni layer and deposed on silica substrate. SiO_2_ is widely used in THz range as optical window being non-absorptive unlike conventional (1–10 Om/cm) silicon. Comparing the simulation results obtained for metasurfaces having periods of 400 μm (a–c) and 600 μm (d–f), one can see that the first one demonstrates a narrow absorptance band when the hemisphere diameter is bigger than 300 μm. In contrast, the metasurface composed of polymer hemispheres with a period of 600 microns is highly absorptive in the wide frequency range 250–600 GHz, when the hemispheres diameter is in the range from 350 to 550 microns.

**Figure 2:**
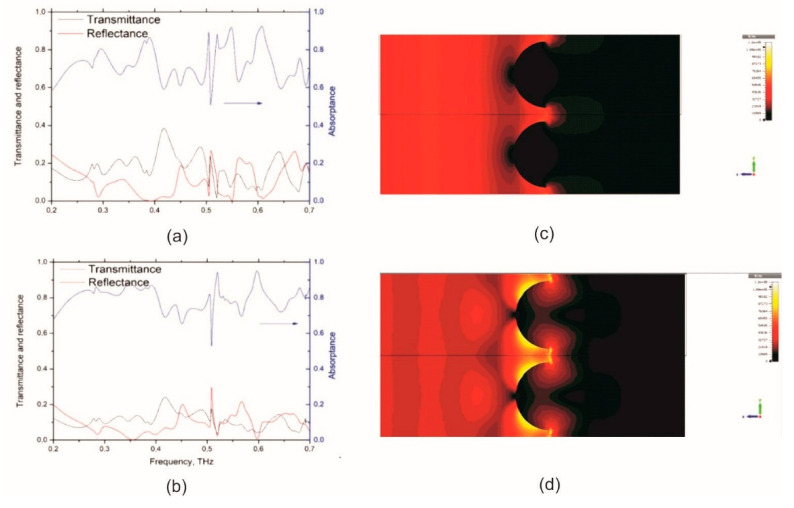
Frequency dependence of the transmittance (*T*), reflectance (*R*) and absorptance (*A*) for (**a**) SiO_2_\polymer hemispheres\Ni and (**b**) SiO_2_\polymer hemispheres\Ni\graphene. (**c**) Distribution of the electric field amplitude at a frequency of 0.6 THz at the surface of the structure SiO_2_\polymer hemispheres\Ni; (**d**) the same for the SiO_2_\polymer hemispheres\Ni\graphene. The following parameters were used in the calculations: the diameter and period of the hemispheres on the SiO_2_ surface are 440 μm and 600 μm, respectively, the sheet conductance of graphene is 1.1 mS, the nickel sheet conductance is 3 mS.

One can see from the spectra presented in Figure 2a that the EM response of the metasurface with the period 600 microns, comprising polymer hemispheres of 440 microns diameter covered with thin metal film, demonstrates a rather good absorption performance showing the average absorptance at the level of 75% and maximum absorptance of as high as 90% at resonance frequencies 0.38, 0.54 and 0.6 THz. It is worth noting that due to pronounced frequency dependence of Ni surface conductance in this frequency range, the SiO_2_\polymer hemispheres\Ni structure is almost 40% transparent in the low-frequency edge of the THz spectra.

On the contrary to the thin Ni metal film, the surface conductivity of graphene is frequency independent in the THz domain [28] (i.e., one may expect that enhancing the metasurface with graphene may results in higher absorptance). Figure 2b shows that placing graphene sheet onto the metasurface enhances absorptance to about 90% in the broad spectral range and >95% at resonant frequencies of 0.52 THz and 0.6 THz. The distribution of the electric field amplitude at a frequency of 0.6 THz is presented in Figure 2c,d, and most clearly illustrates the enhancement effect. One may see that placing the graphene onto the metasurface leads to higher electric field amplitude at the surface of hemispheres and in the gap between the hemispheres indicating the higher absorption ability of graphene-enhanced architecture.

## 3. Materials and Methods

### 3.1. D Printing

In order to fabricate graphene enhanced THz metasurface, we employ a high-resolution layer-by-layer 3D printing technique [29,30,31] with 3D printer having three independent heads. Each head sends out drops of the liquid polymer LUX-Opticlear (Luxexcel, Alpharetta, GA, USA). The lateral size of the drop on the substrate <20 microns. The drops ejected by three heads form a <5 μm thick layer having prescribed lateral structure. As soon as the layer was solidified under UV radiation the next layer is deposited.

To demonstrate the performance of the optimized metasurface, we printed an array of hemispheres with diameter of 300 and 440 μm arranged in the square lattice with a period of 600 μm on the silica substrate, 0.53 mm thick.

### 3.2. Graphene Synthesis and Transfer Fabrication of the Metasurface

We employ CVD graphene synthesized onto 25 μm thick copper foil (Alfa Aesar, Ward Hill, MA, USA, 99.9%) in the Carbolite Gero oven. Prior to the start of the synthesis process, the system was pumped out for 1 h to pressure of 0.1 mbar, then pumped at presence of N_2_/H_2_ (60 sccm) mixture for 1 h, and after that was heated up to the working temperature of 1050 °C at the rate of 20 °C/min. The parameters of the graphene synthesis process are the following: temperature at dynamic regime is 1050 °C, pressure is 4–4.2 mBar, the CH_4_ and H_2_ gas flow is 60 sccm, synthesis time is 120 min.

The graphene sheet synthesized on copper foil was covered with a 200 nm think PMMA layer, which is needed for transfer purposes. The covalent bonding of polymer to carbon atoms in the graphene sheet may result in extra doping of graphene, i.e., to changing its surface conductivity [32]. The typical conductivity of the CVD graphene, which was synthesized on copper foil and then transferred to the dielectric substrate by using PMMA film, is about 1.1 mS [33].

**Figure 3:**
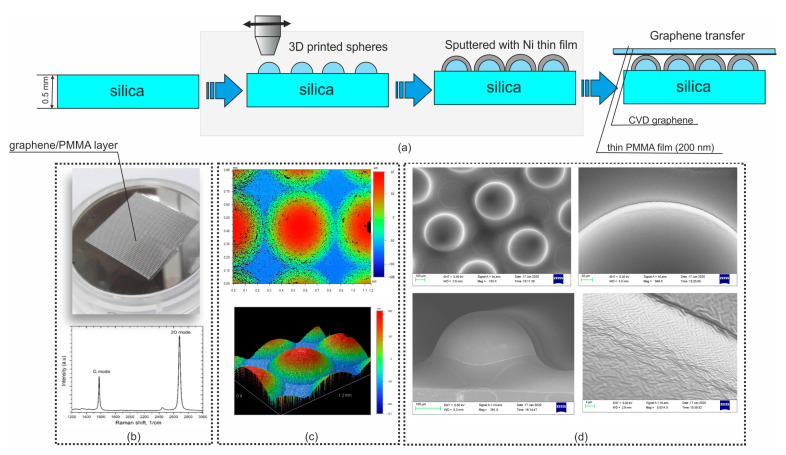
(**a**) Schematic presentation of the graphene enhanced structure fabrication using high-resolution 3D printing. (**b**) Photographic image of the fabricated structure and Raman spectrum of CVD graphene transferred on Si/SiO_2_ (300 nm) substrate for characterization. (**c**) Surface profile of the fabricated structure. (**d**) SEM images of 3D printed polymer hemispheres covered with Ni at different magnifications.

The copper foil was then removed by wet etching in the 30:1:2.5 H_2_O:H_2_O_2_:HCl solution during 2 h. After rinsing in water for one hour the PMMA/graphene bilayer is transferred to surface of the silica substrate with imprinted polymer hemispheres, which were pre-covered with 30 nm thin layer of nickel via either thermal evaporation or magnetron sputtering (see Figure 3a for schematic representation of the metasurface fabrication process and Figure 3b presenting the photo of fabricated structures).

### 3.3. Structural Characterization

The surface topology and roughness of the polymer/Ni 3D printed structure were monitored by the optical profilometer Dektak 6M (Veeco Instruments, Plainview, NY, USA) and by Scanning electron microscopy (SEM-LEO 1550 Gemini, Zeiss, Jena, Germany), as presented in Figure 3c,d, respectively.

The quality of CVD graphene was controlled by Raman spectroscopy, HORIBA XploRA PLUS System, France, at 15 s exposure time at ×100 magnification, using power below 0.8 mW of 532 nm laser for excitation, see Figure 3b. The sharp G peak in the vicinity of 1576 cm^−1^ (FWHM ~21 cm^−1^) and Lorentz shape of 2D peak (peak at 2669 cm^−1^) with FWHM ~30 cm^−1^ indicate (or are inherent in) the single-layer graphene. Furthermore, the absence of D peak in the vicinity of 1360 cm^−1^ emphasizes a high crystallinity of our CVD graphene.

## 4. Experimental Results

**Figure 4:**
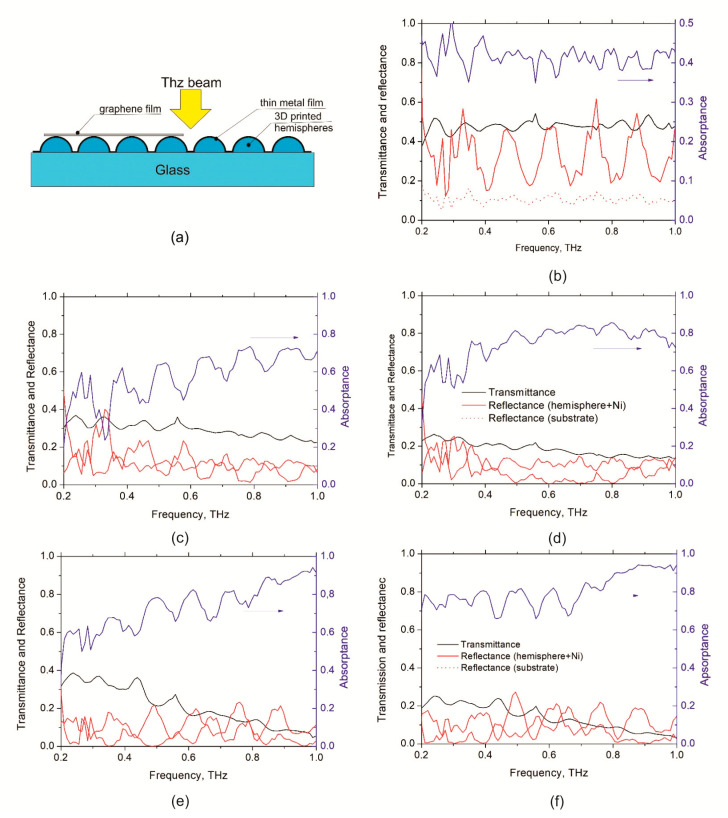
(**a**) Schematic presentation of the experiment. (**b**) Transmittance (*T*), reflectance (*R*) and absorptance (*A*) spectra of the 30 nm thick Ni film deposited on plane SiO_2_ substrate; (**c**,**e**) show *T*, *R* and *A* spectra of SiO_2_\polymer hemispheres\nickel structures composed of hemispheres having diameters 300 μm and 440 μm, respectively; (**d**) and (**f**) show the same spectra for SiO_2_\polymer hemispheres\nickel\graphene structures.

Free space transmittance (*T*) and reflectance (*R*) in THz range was measured using THz time-domain spectrometer (T-Spec, EKSPLA, Vilnius, Lithuania), see the measurement details in [33]. The samples were places by the face side (Ni or graphene/Ni first) towards the 3 mm THz beam treated as a plane wave as shown in Figure 4a.

Figure 4c,e show the measured spectra of the transmittance, reflectance and reconstructed absorptance (*A*) of the fabricated Ni/polymer hemispheres/substrate metasurfaces, whereas Figure 4d,f present the electromagnetic response of graphene/Ni/polymer hemispheres/substrate structures. The spectra for Ni layer, 30 nm thick, deposited on silica substrate are presented for comparison (Figure 4b).

## 5. Discussion and Conclusions

One may see from Figure 4b that the 30 nm thick Ni layer deposited on silica substrate absorbs not more than 40–45% energy of the incident THz radiation. It is worth noting that this result well corresponds to the fact that maximum absorptance of the free-standing metal film does not exceed 50% [34]. However, our experimental and theoretical results show that structuring of the surface can considerably increase absorptance. Specifically, spectra in Figure 2a and Figure 4c,e show that metasurface composed of polymer hemispheres having optimized diameters that arranged in the square lattice with the period of 600 μm and covered 30 nm thick Ni layer can absorb much more than 50% of the incident radiation.

Figure 4c,e and Figure 4d,f show *T*, *R* and *A* of the metasurfaces without and with graphene, respectively. It is important to note that metasurfaces without graphene demonstrate pronounced frequency dependence of the *T*, *R*, and *A* having absorptance approaching 80–90% only at frequencies close to 1 THz, as it is expected from the modeling results (see Figure 1). The placing graphene sheet onto the SiO_2_\polymer hemispheres\Ni metasurface allows us to increase absorptance in the whole frequency range (Figure 2b and Figure 4d,f for modeling and experimental data correspondently). Moreover, the metasurface having hemispheres’ diameter of 440 microns demonstrates absorptance of above 80% at 0.2–1 THz.

One can conclude that replacing flat substrate with 3D structured one can increase the absorptance level from 50% to 80–90%. The presence of an additional graphene layer in the system makes it possible to significantly change the dispersion of the electromagnetic response and increase the overall absorption level, particularly at low frequencies.

## Data Availability

The raw data are available upon request from authors.

## References

[1] Kuzhir P., Paddubskaya A., Volynets N., Batrakov K., Kaplas T., Lamberti P., Kotsilkova R., Lambin P. (2017). The main principles of passive devices based on graphene and carbon films in microwave—THz frequency range. J. Nanophotonics.

[2] Efetov D.K., Shiue R.-J., Gao Y., Skinner B., Walsh E.D., Choi H., Zheng J., Tan C., Crosso G., Peng C. (2018). Fast thermal relaxation in cavity-coupled graphene bolometers with a Johnson noise read-out. Nat. Nanotechnol..

[3] Blaikie A., Miller D., Alemán B.J. (2019). A fast and sensitive room-temperature graphene nanomechanical bolometer. Nat. Commun..

[4] Watts C.M., Liu X., Padilla W.J. (2012). Metamaterial electromagnetic wave absorbers. Adv. Mater..

[5] Dhillon S.S., Vitiello M.S., Linfield E.H., Davies A.G., Hoffmann M.C., Booske J., Paoloni C., Gensch M., Weightman P., Williams G.P. (2017). The 2017 terahertz science and technology roadmap. J. Phys. D Appl. Phys..

[6] Meisak D., Gurnevich E., Plyushch A., Bychanok D., Georgiev V., Kotsilkova R., Kuzhir P. (2020). Robust design of compact microwave absorbers and waveguide matched loads based on DC-conductive 3D-printable filament. J. Appl. Phys. D.

[7] Chung B.-K., Chuah H.-T. (2003). Modeling of RF absorber for application in the design of anechoic chamber. Prog. Electromagn. Res..

[8] Ren J., Yin J.Y. (2018). 3D-Printed low-cost dielectric-resonator-based ultra-broadband microwave absorber using carbon-loaded acrylonitrie butadiene styrene polymer. Materials.

[9] Kim D.S., Kim D.J., Kim D.H., Hwang S., Jang J.H. (2012). Simple fabrication of an antireflective hemispherical surface structure using a self-assembly method for the terahertz frequency range. Opt. Lett..

[10] Batrakov K., Kuzhir P., Maksimenko S., Paddubskaya A., Voronovich S., Lambin P., Kaplas T., Svirko Y. (2014). Flexible transparent graphene/polymer multilayers for efficient electromagnetic field absorption. Sci. Rep..

[11] Wu Z.S., Ren W., Gao L., Zhao J., Chen Z., Liu B., Tang D., Yu B., Jiang C., Cheng H.M. (2009). Synthesis of Graphene Sheets with High Electrical Conductivity and Good Thermal Stability by Hydrogen Arc Discharge Exfoliation. ACS Nano.

[12] Gosling J.H., Makarovsky O., Wang F., Cottam N.D., Greenaway M.T., Patanè A., Wildman R.D., Tuck C.J., Turyanska L., Fromhold T.M. (2021). Universal mobility characteristics of graphene originating from charge scattering by ionised impurities. Commun. Phys..

[13] Novoselov K.S., Geim A.K., Morozov S.V., Jiang D., Katsnelson M.I., Grigorieva I.V., Dubonos S.V., Firsov A.A. (2005). Two-dimensional gas of massless Dirac fermions in graphene. Nature.

[14] Docherty C.J., Lin C.T., Joyce H.J., Nicholas R.J., Herz L.M., Li L.J., Johnston M.B. (2012). Extreme sensitivity of graphene photoconductivity to environmental gases. Nat. Commun..

[15] Tasolamprou A.C., Koulouklidis A.D., Daskalaki C., Mavidis C.P., Kenanakis G., Deligeorgis G., Viskadourakis Z., Kuzhir P., Tzortzakis S., Kafesaki M. (2019). Experimental demonstration of ultrafast THz modulation in a graphene-dased thin film absorber through negative photoinduced conductivity. ACS Photonics.

[16] Arik K., Abdollahramezani S., Khavasi A. (2017). Polarization insensitive and broadband terahertz absorber using graphene disks. Plasmonics.

[17] Li X., Cai W., An J., Kim S., Nah J., Yang D., Piner R., Velamakanni A., Jung I., Tutuc E. (2009). Large-area synthesis of high-quality and uniform graphene films on copper foils. Science.

[18] Lin W.-H., Chen T.-H., Chang J.-K., Taur J.-I., Lo Y.-Y., Lee W.-L., Chang C.-S., Su W.-B., Wu C.-I. (2014). A direct and polymer-free method for transferring graphene grown by chemical vapor deposition to any substrate. ACS Nano.

[19] Choi J., Kim H.J., Wang M.C., Leem J., King W.P., Nam S. (2015). Three-dimensional integration of graphene via swelling, shrinking and adaptation. Nano Lett..

[20] Lanza M., Bayerl A., Gao T., Porti M., Nafria M., Jing G.Y., Zhang Y.F., Liu Z.F., Duan H.L. (2012). Graphene-coated atomic force microscope tips for reliable nanoscale electrical characterization. Adv. Mater..

[21] Román-Manso B., Figueiredo F.M., Achiaga B., Barea R., Pérez-Coll D., Morelos Gómez A., Terrones M., Osendi M.I., Belmonte M., Miranzo P. (2016). Electrically functional 3D architectured graphene/SiC composites. Carbon.

[22] Kaplas T., Sharma D., Svirko Y. (2012). Few-layer graphene synthesis on a dielectric substrate. Carbon.

[23] Wang H., Hsu A.L., Palacios T. (2012). Graphene Electronics for RF Applications. IEEE Microw. Mag..

[24] Chen H., Lu W.B., Liu Z.G., Geng M.Y. (2020). Microwave programmable graphene metasurface. ACS Photonics.

[25] Zhang Y., Li T., Chen Q., Zhang H., O’Hara J.F., Abele E., Taylor A.J., Chen H.T., Azad A.K. (2016). Independently tunable dual-band perfect absorber based on graphene at mid-infrared frequencies. Sci. Rep..

[26] Paddubskaya A., Demidenko M., Batrakov K., Valušis G., Kaplas T., Svirko Y., Kuzhir P. (2019). Tunable perfect THz absorber based on the stretchable ultrathin carbon-polymer bilayer. Materials.

[27] Andreev V.G., Vdovin V.A., Voronov P.S. (2003). An experimental study of millimeter wave absorption in thin metal films. Tech. Phys. Lett..

[28] Buron J.D., Pizzocchero F., Jessen B.S., Booth T.J., Nielsen P.F., Hansen O., Hilke M., Whiteway E., Jepsen P.U., Boggild P. (2014). Electrically continuous graphene from single crystal copper verified by terahertz conductance spectroscopy and micro four-point probe. Nano Lett..

[29] Assefa B.G., Saastamoinen T., Biskop J., Kuittinen M., Turunen J., Saarinen J. (2018). 3D printed plano-freeform optics for non-coherent discontinuous beam shaping. Opt. Rev..

[30] Assefa B.G., Saastamoinen T., Pekkarinen M., Biskop J., Nissinen V., Kuittinen M., Turunen J., Saarinen J. (2019). Realizing freeform optics using 3D-printer for industrial based tailored irradiance distribution. OSA Contin..

[31] Assefa B.G., Pekkarinen M., Partanen H., Biskop J., Turunen J., Saarinen J. (2019). Imaging-quality 3D-printed centimeter-scale lens. Opt. Express.

[32] Reina A., Jia X., Ho J., Nezich D., Son H., Bulovic V., Dresselhaus M.S., Kong J. (2009). Large area, few-layer graphene films on arbitrary substrates by chemical vapor deposition. Nano Lett..

[33] Batrakov K., Kuzhir P., Maksimenko S., Volynets N., Voronovich S., Paddubskaya A., Valusis G., Kaplas T., Svirko Y., Lambin P. (2016). Enhanced microwave-to-terahertz absorption in graphene. Appl. Phys. Lett..

[34] Jackson J.D. (1998). Classical Electrodynamics.

